# Essential trauma management training: addressing service delivery needs in active conflict zones in eastern Myanmar

**DOI:** 10.1186/1478-4491-7-19

**Published:** 2009-03-03

**Authors:** Allison J Richard, Catherine I Lee, Matthew G Richard, Eh Kalu Shwe Oo, Thomas Lee, Lawrence Stock

**Affiliations:** 1 Keck School of Medicine, Los Angeles, CA, USA; 2 Global Health Access Program, Mae Sot, Tak, Thailand; 3 David Geffen School of Medicine, University of California at Los Angeles, Los Angeles, CA, USA; 4 Karen Department of Health and Welfare, Mae Sot, Tak, Thailand

## Abstract

**Introduction:**

Access to governmental and international nongovernmental sources of health care within eastern Myanmar's conflict regions is virtually nonexistent. Historically, under these circumstances effective care for the victims of trauma, particularly landmine injuries, has been severely deficient. Recognizing this, community-based organizations (CBOs) providing health care in these regions sought to scale up the capacity of indigenous health workers to provide trauma care.

**Case description:**

The Trauma Management Program (TMP) was developed by CBOs in cooperation with a United States-based health care NGO. The goal of the TMP is to improve the capacity of local health workers to deliver effective trauma care. From 2000 to the present, international and local health care educators have conducted regular workshops to train indigenous health workers in the management of landmine injuries, penetrating and blunt trauma, shock, wound and infection care, and orthopedics. Health workers have been regularly resupplied with the surgical instruments, supplies and medications needed to provide the care learnt through TMP training workshops.

**Discussion and Evaluation:**

Since 2000, approximately 300 health workers have received training through the TMP, as part of a CBO-run health system providing care for approximately 250 000 internally displaced persons (IDPs) and war-affected residents. Based on interviews with health workers, trauma registry inputs and photo/video documentation, protocols and procedures taught during training workshops have been implemented effectively in the field. Between June 2005 and June 2007, more than 200 patients were recorded in the trauma patient registry. The majority were victims of weapons-related trauma.

**Conclusion:**

This report illustrates a method to increase the capacity of indigenous health workers to manage traumatic injuries. These health workers are able to provide trauma care for otherwise inaccessible populations in remote and conflicted regions. The principles learnt during the implementation of the TMP might be applied in similar settings.

## Introduction

The government of Myanmar directs less than 3% of its budget annually towards health care, resulting in scant services for its people [1]. In the border regions, access to both governmental and international nongovernmental sources of health care is worse than in the rest of Myanmar. This is largely a result of civil conflict and government restrictions that have persisted for decades. While much attention is rightfully paid to the problem of infectious diseases and a failing health care system in Myanmar, attention must also be paid to the widespread use of landmines. The 2007 *Landmine monitor report *identifies Myanmar as one of the few countries experiencing an increase in the number of landmine casualty rates in 2006, reporting 243 new casualties, up from 231 in 2005 [2]. These statistics, however, likely reflect severe underreporting, as most injuries occur in areas where data are not routinely collected. Mortality surveys conducted in an eastern Myanmar conflict zone in 2002 demonstrated that 4% of all deaths were attributable to landmines [3].

The reason for these high injury and mortality rates is multifactorial. Although landmines are used in combat by both government forces and their adversaries, the United Nations Special Rapporteur on Human Rights stated his concerns about the use of landmines against civilians in his report to the United Nations in 2007: "Among the most appalling features of the military campaign in ethnic areas is the disproportionate effect on civilian populations..." [4]. The Karen Human Rights Group has documented villagers' reports of "atrocity demining", whereby the Myanmar Army forces villagers to walk in front of soldiers as human minesweepers [5]. In addition, the Thailand Burma Border Consortium stated that mines are often placed near rice fields to prevent villagers from cultivating the land and to aid in the displacement of these civilian populations [6]. Finally, a survey of human rights violations in eastern Myanmar found that households that were forcibly displaced were four times more likely to have a household member become a landmine victim [7].

Landmines in these areas usually require only 6 kg of pressure to be triggered, ensuring that even a child or animal can cause an explosion. For the significant proportion of adults and children who survive the initial blast, rapid access to care is crucial. Beyond initial stabilization, higher-level care is essential, as many survivors require critical actions, including amputation.

Yet for landmine victims in conflict areas of eastern Myanmar, there is little or no access to care. The Myanmar government's so-called "Four Cuts Policy", which aims to cut off the supply of food, funding, information and recruits to ethnic minority insurgents, also prevents access to government and international forms of humanitarian assistance. By 2004 there were more than 500 000 internally displaced persons (IDPs) in eastern Myanmar, living in these areas with virtually no access to hospitals, physicians or nurses [6].

In response to these needs, community-based organizations (CBOs) have mobilized to address the most pressing health problems. Two organizations involved in trauma care in eastern Myanmar are the Karen Department of Health and Welfare (KDHW), and the Backpack Health Worker Teams (BPHWT). KDHW is the health department of the Karen National Union, the Karen State (Eastern Myanmar) government-in-exile of the ethnic Karen people. KDHW manages 33 mobile clinics providing care for more than 100 000 internally displaced persons (IDPs) and war-affected residents of Karen State. The clinics are mobile in the sense that they are based in bamboo structures and can be moved quickly in case of attack. Five to ten health workers staff each clinic. BPHWT formed in 1998 to deliver health care services to the most remote areas within the conflict zones of eastern Myanmar. BPHWT is a multiethnic organization (Karen, Karenni, Mon and Shan) that has 90 teams of three to five health workers per team providing care for more than 150 000 IDPs. These mobile teams serve more unstable areas, where it would be impossible to have even semipermanent clinics.

The 711 KDHW and BPHWT health workers are a diverse group. They range in age from 19 to 55 years, 54% male and 46% female. They have received training from a variety of sources including KDHW, BPHWT, IDP camps in Myanmar, refugee camps in Thailand and Mao Tao Clinic (MTC). MTC was established in 1988 by Dr Cynthia Maung in Mae Sot, Thailand, and is the largest training and treatment centre for exiles who have fled to Thailand from Myanmar, yet who are not living under refugee status. Training for a health worker ranges from 4 to 18 months and includes intensive training in basic primary care, infectious disease, maternal child care, first aid and public health. A subset of these health workers returns to the Thai border every six months to receive further training, to exchange data and to resupply.

Since the late 1990s, health care leaders have worked to improve the capacity of health workers trained in trauma care to augment the services provided by KDHW and BPHWT, which has subsequently developed into a more formal programme, the Trauma Management Program (TMP). Although the impetus to establish the TMP was the prevalence of conflict-related trauma due to landmine injuries, skills learned in the trauma courses also apply to injuries incurred by gunshot wounds, stab wounds, blunt trauma, falls and environmental injuries.

We describe the development of a trauma management programme to scale up the number and skills of community health workers to address the health care needs of landmine injury victims. We describe the training programme, including curriculum, training workshops, personnel and resource utilization. We also describe outcomes of training and provide trauma victim data.

## Case description

The TMP had as its predecessor the War Casualty Management Training Course (1993–1996), run by the Trauma Care Foundation (TCF)/Tromsoe Mine Victim Resource Center, as well as training sessions lead by individual trauma care experts. Beginning in 2000, a four-to-six-day trauma course for health workers was established by the Global Health Access Program (GHAP) in conjunction with KDHW to teach basic competences in caring for trauma victims. GHAP is a United States-based, non-profit, nongovernmental organization (NGO) that provides health-related technical assistance and capacity building for CBOs. The course has occurred twice a year for the last eight years and has evolved over time. In the last three years, Australian Aid International (AAI), an Australia-based health care and disaster assistance NGO, has partnered with GHAP and KDHW in the trauma training workshops.

Class composition of approximately 30 students has been two thirds health workers without prior trauma training and one third with prior training and experience in trauma management. KDHW leaders have selected student participants with the goal of creating integrated trauma teams of experienced and less-experienced health workers. Course instructors have included GHAP and AAI volunteer physicians, registered nurses, nurse practitioners and pre-hospital care personnel, together with the more experienced trauma health workers. Volunteer physicians have included emergency medicine physicians, general surgeons and orthopaedic surgeons. A training-of-trainers programme is embedded in the current course, in which the experienced trauma health workers serve as mentors, small group leaders and lecturers during the biannual course, thus increasing their capacity as trainers within their health care system.

The curriculum covers the evaluation and management of the trauma victim, with an emphasis on resuscitation, stabilization, recognition and management of shock, wound care and prevention of infection, sepsis and organ failure. The trauma course content has drawn from resources developed by the TCF, the International Committee of the Red Cross, Dr Maurice King's series of books on primary surgical care and a variety of other authoritative sources. The course focuses on the early and aggressive management of limb injuries, including control of bleeding, wound care, fasciotomy, amputation, fracture and dislocation management, splinting and casting. Other skills taught include: suturing; anaesthesia and analgesia; preoperative, operative and postoperative care; monitoring, hygiene and psychological care of the trauma patient; rehabilitation; basic and advanced/surgical airway; tube thoracostomy; venous cut down; nasogastric and urine catheter use; intravenous fluid therapy; blood typing; and blood transfusion. A short, focused lecture followed by a clinical activity has been the typical teaching pattern, within a three-hour teaching block each morning and each afternoon. Activities include role-playing, skills labs and case reviews. Each course has been designed to cover the basic core content, but with some new concepts added to each subsequent course for the benefit of returning experienced health workers. The health workers are assessed throughout the course by the faculty. A pre-course and post-course written quiz is administered on core concepts. Skills during role-playing trauma drills and skill labs are observed and feedback is given to student health workers throughout the course in real time.

In the last 12 months, senior trauma health workers have developed advanced and basic trauma curricula for field training for the larger number of health workers who remain in the field and make up most of the health care infrastructure. In addition, KDHW and BPHWT also provided first-responder health training for local villagers in their respective target populations ("Village Health Workers" or VHWs). A total of 333 VHWs have received training from one week's to two months' duration in first aid and primary care. VHWs live in the villages where trauma often occurs, and training this group is under way as a crucial link in the trauma chain of survival.

Health workers trained in trauma care work in their assigned clinics or backpack teams. In addition, specialized teams of these health workers based at clinics can be "activated" or called to a village in the case of a trauma patient who cannot be transported. Health workers often function in remote jungle and village settings, and thus are trained to be members of mobile and self-reliant teams. These teams consist of experienced and less-experienced health workers in each geographical area of coverage, a practice that fosters teamwork and mentorship and results in the transition from junior to senior trauma health worker status over time. Senior health workers teach, supervise and informally evaluate junior health workers within each field team.

The TMP provides trauma teams with a standard set of supplies, including stethoscopes, surgical instruments, headlamps, files, amputation saws and modified tourniquets. Other basic supplies include gloves; gauze; ace wraps; tape; suture; tubing for airways and chest tubes; irrigation supplies; injection and IV supplies; rapid diagnostic kits for HIV and blood typing; blood transfusion supplies; and antiseptics. Medications include basic oral and IV antibiotics, analgesics and anaesthetics.

The TMP has created data collection tools to facilitate the process of patient care, resource management and trauma patient outcomes analysis. Data collection began in June 2005. Health workers complete each form in the field while they are conducting patient care. Data fields include patient name; age; sex; date and time of injury; mechanism of injury; region of body injured; date and time of health worker arrival to patient and departure from patient; treatment given; referral information; and survival information. Trauma health workers are not activated for deceased victims; any patient who died prior to health worker arrival is excluded from the Trauma Care Registry. Included are all other patients whom the trauma team was activated to see, including patients with blunt trauma, penetrating trauma and blast injuries. Survival is defined as a patient who had signs of life on trauma health worker arrival and was considered highly likely to survive this injury upon health worker departure. Unstructured interviews with health workers, trauma registry inputs and photo/video documentation were all used to determine what trauma procedures were performed in the field.

About 40 new health workers per year have received trauma training since 2000 in essential trauma-management skills. About 10 specific health workers have attended all or most workshops during this same period. The majority of the trauma course students are from Karen State. Health workers from Mon, Karenni and Shan State have also participated. Real-time course observations and feedback by trauma course faculty to health worker students have been the main measure of student comprehension of course content.

In 2007, after the formation of field curricula, trauma health workers had conducted four Village Health Worker First Aid Training Courses, and one Basic Field Trauma Health Worker Course. Limitations to expansion of training include security constraints in moving health workers from one area to another within the conflict zone of Karen State and the costs of training.

From June 2005 to June 2007, these trauma health workers provided services to more than 200 patients recorded in the trauma registry. Although adequate comparison data upon which to judge efficacy are lacking, the data collected can serve as an estimate of what types of injuries are being seen and what type of care is being given. Demographic characteristics of the population are shown in Table 1. The majority of trauma victims were young (mean age, 30 years) and male (89%).

**Table 1:** Demographic characteristics of the study population (N = 183)

**Variable**	**Male**	**Female**	**Total**
	
	N(%)	N(%)	N(%)
**Gender**			

Male	-	-	163 (89)

Female	-	-	20(11)

**Age**			

< 18	12(7)	4(20)	16(9)

19–24	37(23)	5(25)	42(23)

25–44	88(54)	3(15)	91(50)

> 45	20(12)	6(30)	26(14)

Not recorded	6(4)	2(10)	8(4)

**Cause of injury**			

Landmine	76(47)	4(20)	80(44)

Gunshot	39(24)	3(15)	42(23)

Fall from tree	6(4)	-	6(3)

Hit by tree	3(2)	-	3(2)

Cut wound	8(5)	-	8(4)

Burn	1(1)	4(20)	5(3)

Animal attack	9(6)	-	9(5)

RPG/mortar	2(1)	-	2(1)

Stab wound	4(2)	3(15)	7(4)

Other	15(9)	6(30)	21(11)

**Outcome**			

Survived	147(90)	20(100)	167(91)

Expired	16(10)	-	16(9)

**Wait, in days, for medic arrival**			

*Mean*	*2.35*	*1.28*	*2.23*

A wide variety of trauma mechanisms were reported, including weapons-related, accident and animal attack. The majority (72%), however, were a result of weapons-related trauma. Landmine injury was the most common type, followed by gunshot wounds. A few additional cases of stab and mortar/RPG injury were reported. Of all patients receiving care by the health workers, the vast majority (91%) survived and were alive at the time of last contact.

Sixteen patients (9%) treated by health workers ultimately expired as a result of their injuries. Characteristics of patients who died are shown in Table 2. Compared to the overall population, patients who died were more likely to have suffered weapons-related trauma (94% of injuries). Landmine and gunshot wounds accounted for 15 deaths, with one patient dying after falling from a tree. All the deceased patients were male, with ages similar to the overall population. Compared with survivors, those who died had a much higher rate of injury to the head and torso, the same as would be expected in a high-resource medical care system.

**Table 2:** Characteristics of subjects who did not survive (N = 16)

**Variable**	**N(%)**
**Gender**	

male	16(100)

Female	-

**Age *Mean(SD)***	

< 18	-

19–24	5(31.25)

25–34	8(50.00)

35–44	3(18.75)

45–54	-

55–64	-

65–74	-

75–84	-

**Cause of Injury**	

Landmine	8(50)

Gunshot	7(43.75)

Fall from tree	1(6.25)

Hit by tree	-

PPH	-

Abscess	-

Cut wound	-

Burn	-

Animal attack	-

RPG/mortar	-

Severe malaria	-

Stab wound	-

Other	-

Don't know	-

**Wait time for medic**	

0 days	8(61.54)

1–5 days	4(30.77)

6–10 days	-

11–20 days	-

21–30 days	-

> 31 days	1(7.69)

A wide spectrum of treatment modalities was used in the care of trauma victims. Evidence acquired through unstructured interviews with health workers, trauma registry inputs and photo/video documentation suggests that procedures taught during training workshops were implemented effectively in the field. In the treatment of severe extremity injuries, fasciotomy and amputation were commonly performed. Ketamine was typically used for procedural sedation and intravenous fluids were used in resuscitation before, during and after the procedure. Patient assessment, monitoring and basic airway skills were routinely used. Advanced airway and tube thoracostomy skills were rarely used. Blood transfusions were performed for haemorrhagic shock. Wound care was performed and antibiotics (intravenous and oral) were frequently administered. Splinting was performed with either plaster or bamboo.

## Discussion and evaluation

Trauma continues to be a significant source of morbidity and mortality in the conflict regions of Eastern Myanmar. One in 50 households reports exposure to combat-related violence, with landmine death or injury affecting 13.3 per 10 000 population annually [7]. In addition, Hougen et al. interviewed 188 refugees from Myanmar living in Thailand, and found that 23 were landmine survivors, the majority civilians [8].

In this report, we describe the development of a trauma-training programme by and for a CBO of IDPs in partnership with a health care NGO. We demonstrate that mobile health workers in a low-resource setting, with no immediate access to hospitals or other well-resourced referral centres, can be trained and equipped to treat life-threatening injuries. Overall, trauma victims treated by health workers survived in 91% of cases. Of landmine patients, the largest group, 90% survived initial treatment and were considered stable at the time of last health worker contact. Although we have no adequate comparison data specific to this setting and these conditions, we believe that these numbers are notable, considering that treatment was provided in a jungle conflict zone, with limited shelter, no electricity, and equipment limited to that which could be carried on foot to reach victims who might be several hours' or days' hike distant. Additionally, health workers worked in a hostile environment where they themselves were at risk of becoming victims of conflict-related trauma.

Based on unstructured interviews with health workers, data gathered and faculty observations, we believe the curriculum and training provided in the trauma workshops has been helpful in upgrading the skills and number of trauma health workers in eastern Myanmar. The curriculum and course emphasis have been adapted over time due to health worker feedback and data and continue to better reflect the needs of the trauma health workers.

We lack data on trauma mortality prior to the initiation of the TMP, making it is impossible to quantify the health outcome benefit with our data. However, based on available documentation, victims of trauma are now receiving care that was not widely available prior to the TMP.

There are a number of limitations to this report. First, data gathering was performed using standardized forms, but in some cases, documentation was incomplete. Also, given the difficult and unpredictable conditions in which the health workers work, it is likely that some trauma patients may have been treated, but not recorded in the trauma registry. Second, we cannot establish with certainty the degree to which the TMP has improved outcomes, since no data are available prior to programme implementation.

## Conclusion

As trauma is increasingly recognized as a major cause of morbidity and mortality in the developing world, effective health worker trauma training has increasing applicability for other conflict, post-conflict and low-accessibility areas. This report illustrates the development and implementation of a health worker-run trauma care training and system by a community-based organization partnering with an NGO. Finally, in interviews, health workers report that skills and knowledge acquired through the TMP have imbued them with confidence and a sense of empowerment in situations that once seemed hopeless.

## Competing interests

The authors declare that they have no competing interests.

## Authors' contributions

AR contributed to conception and design of the manuscript and analysis and interpretation of data. CL participated in the conception and design of the manuscript and acquisition of data. MR assisted in composing the manuscript. EK made contributions in data collection and critical revision of the final manuscript for intellectual content. TL participated in the final review of the manuscript. LS conceived of the project and participated in the design and drafting of the manuscript. All authors read and approved the final manuscript.
